# Hepatitis E Virus Genotype 4, Denmark, 2012

**DOI:** 10.3201/eid2001.130600

**Published:** 2014-01

**Authors:** Sofie Midgley, Hanne Thang Vestergaard, Camilla Dalgaard, Lone Enggaard, Thea Kølsen Fischer

**Affiliations:** Statens Serum Institut, Copenhagen, Denmark

**Keywords:** hepatitis E virus, genotype, autochthonous, emergence, zoonoses, Denmark, viruses

**To the Editor:** Hepatitis E virus genotype 4 (HEV4) is most commonly reported in China and Japan; it has primarily been categorized as a zoonotic virus because it has been found in humans and several other animal species (*1*). In Europe, HEV4 has been identified in 6 countries, in humans and other animals. The first cases of HEV4 infection in a human and an animal (pig) were detected through strain surveillance activities in Germany (*2*) and Belgium (*3*), respectively. In May 2009, France reported an isolated case of HEV4 infection in a human; however, the source of infection could not be determined. Consumption of contaminated food was deemed unlikely, but receipt of contaminated blood during transfusion was possible (*4*). In 2011, an outbreak of HEV4 in France was associated with consumption of figatelli, a liver sausage that is traditionally consumed uncooked (*5*). Also in 2011, an outbreak in Italy was reported, in which contaminated water was suggested as a possible source of infection (*6*). HEV4 has also been detected in a patient in the United Kingdom. This patient, however, had just returned from India, so the infection was associated with travel (*7*). Transmission was suspected to be zoonotic because the sequence was most closely related to isolates from swine in India.

From January 2010 through December 2012, the Department of Microbiological Diagnostics and Virology of the Statens Serum Institut received 1,112 samples from 823 patients with clinically suspected hepatitis for HEV diagnostic serologic and PCR testing. Blood samples were tested for HEV IgM and IgG by use of the DS-EIA-ANTI-HEV-M and DS-EIA-ANTI-HEV-G kits (DSI, Saronno, Italy) according to the manufacturer’s instructions. Diagnostic real-time reverse transcription PCR (RT-PCR) amplifying a 79-bp fragment of open reading frame 3 (*8*) was conducted by using a QIAGEN OneStep RT-PCR Kit (Hilden, Germany). Samples from 59 (7%) patients were HEV positive according to serologic testing; of these, 20 (34%) were positive for HEV RNA according to RT-PCR.

Genotyping was conducted by amplifying 804 bp of open reading frame 2. By sequencing of PCR products, 13 (65%) samples were successfully genotyped; 3 in 2010, 7 in 2011, and 3 in 2012. In 2010 and 2011, HEV genotypes 3 and 1, respectively, were detected in 5 patients. In 2012, a total of 3 patients were infected with HEV genotype 4. 

The 3 sequences obtained in this study (GenBank accession nos. KC928081–KC928083) were subjected to phylogenetic analysis, along with reference sequences that included the sequences from the France and Italy outbreaks and the strain from the HEV4 virus identified in a pig in Belgium in 2008. All sequences were aligned by using the Simmonic sequence editor (*9*). Phylogenetic trees were constructed by using MEGA5 (*10*) with the maximum-likelihood method and the Jukes-Cantor algorithm with 500 bootstrap replicates.

Of the 3 patients from Denmark, the HEV4 sequence from 1 patient (March 2012) resembled the sequence from Italy, and these 2 strains grouped together with strains from humans and other animals from China (Figure). This patient reported having traveled to China; thus, zoonotic infection acquired while abroad is suggested. The other 2 patients from Denmark (June 2012) reported no travel history, and the viruses detected in these patients were almost identical; only 2 nt differences were found. No epidemiologic or geographic link connected these 2 patients. The only link was the date of sample collection. Both patients were ill during the summer, which suggests possible consumption of undercooked, or raw, contaminated food as the source of infection. The sequences from these 2 patients were most closely related to the sequences from patients involved in the outbreaks in France (Figure). These sequences all form a group with the HEV4 virus identified in the pig in Belgium in 2008, thereby suggesting a zoonotic origin.

**Figure:**
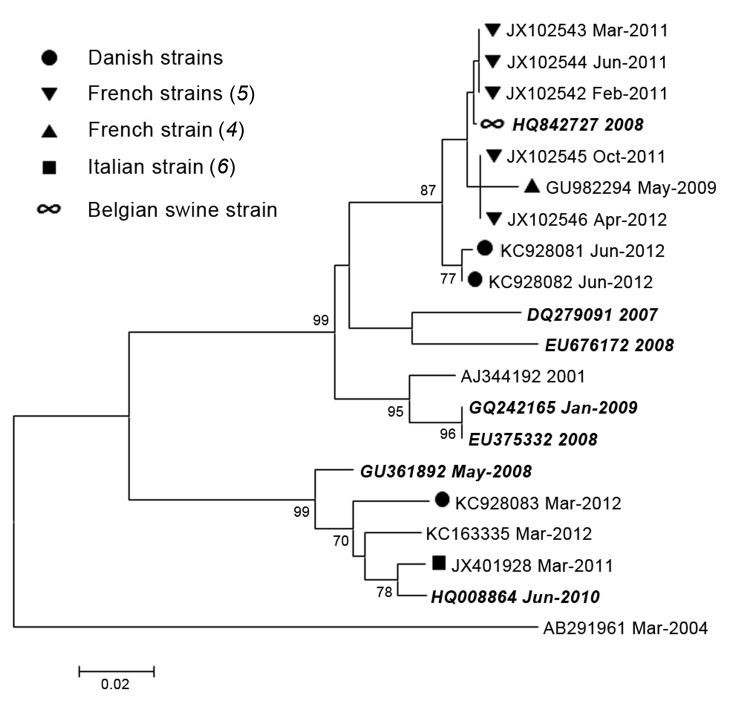
Maximum-likelihood phylogenetic analysis of hepatitis E virus genotype 4. Reference sequences are identified by their GenBank accession numbers; animal strains are indicated in ***boldface italics***. Months (when available) and years of sample collection are indicated after the sequence names. Countries from which strains were isolated are indicated by symbols. Strain AB291961 is a human genotype 3 reference strain included as an outgroup. Scale bar indicates nucleotide substitutions per site.

Because Statens Serum Institut is the only laboratory in Denmark that offers diagnostic testing for HEV, we consider our national surveillance to be fairly complete. Prospective surveillance will show whether HEV4 becomes established within Denmark. To date, HEV4 has not been detected in animal populations in Denmark. In China, similarity of HEV4 data between strains from humans and other animals in the same geographic areas was high, which is highly suggestive of zoonotic transmission (*3*). Because of the rare detection of HEV4 in Europe, these types of data are not yet available for European countries. However, the close phylogenetic relationship between the strains from humans in Denmark and France and the strain from the pig in Belgium suggests a zoonotic origin for this genotype in these countries. This suggestion is further supported by the fact that some of the strains from France were associated with the consumption of pork liver sausage. 

The emergence of autochthonous HEV4 infection in human populations in 4 European countries, and its detection in different years (2006/2007, 2008, 2009, 2011, and 2012), suggests that this genotype may be established in Europe. Thus, for the purpose of ensuring HEV4 detection, diagnostic and genotyping methods should be evaluated.
